# The VLPFC versus the DLPFC in Downregulating Social Pain Using Reappraisal and Distraction Strategies

**DOI:** 10.1523/JNEUROSCI.1906-20.2020

**Published:** 2021-02-10

**Authors:** Jun Zhao, Licheng Mo, Rong Bi, Zhenhong He, Yuming Chen, Feng Xu, Hui Xie, Dandan Zhang

**Affiliations:** ^1^School of Psychology, Shenzhen University, Shenzhen 518060, China; ^2^Institute of Brain and Psychological Sciences, Sichuan Normal University, Chengdu 610066, China; ^3^Shenzhen Yingchi Technology Company, Ltd, Shenzhen 518057, China; ^4^Shenzhen-Hong Kong Institute of Brain Science, Shenzhen 518060, China; ^5^Shenzhen Institute of Neuroscience, Shenzhen 518060, China

**Keywords:** dorsolateral prefrontal cortex, emotional regulation, social exclusion, social pain, TMS, ventrolateral prefrontal cortex

## Abstract

The dorsolateral prefrontal cortex (DLPFC) and ventrolateral PFC (VLPFC) are both crucial structures involved in voluntary emotional regulation. However, it remains unclear whether the functions of these two cortical regions that are involved in emotional regulation, which are usually active in non-social situations, could be generalized to the regulation of social pain as well. This study employed transcranial magnetic stimulation (TMS) to examine the causal relationship between the DLPFC/VLPFC and the emotional regulation of social pain via distraction and reappraisal. Ninety human participants (45 males and 45 females) initially underwent either active (DLPFC/VLPFC, *n* = 30/30) or sham (vertex, *n* = 30) TMS sessions. Participants were then instructed to use both distraction and reappraisal strategies to downregulate any negative emotions evoked by social exclusion pictures. Convergent results of the subjective emotional rating and electrophysiological indices demonstrated that: (1) both the DLPFC and VLPFC highly facilitate the downregulation of affective responses caused by social exclusion, revealing a causal role of these lateral PFCs in voluntary emotional regulation of both non-social and social pain; and (2) these two cortical regions showed relative functional specificity for distraction (DLPFC) and reappraisal (VLPFC) strategies, which helps to refine the cortical targeting of therapeutic protocols. In addition, the TMS effect was sustainable for at least 1 h, showcasing the potential feasibility of using this method in clinical practice. Together, these findings provide cognitive and neural evidence for the targeting of the VLPFC and/or the DLPFC to improve emotional regulation abilities, especially in social contexts.

**SIGNIFICANCE STATEMENT** This study aimed to examine the role of the dorsolateral prefrontal cortex (DLPFC) and ventrolateral PFC (VLPFC) in emotional regulation, particularly in response to social pain through the use of distraction and reappraisal strategies, as this is a relatively underexplored area of inquiry. This study makes a significant contribution to the literature because our results provide novel empirical information on the role of these cortical structures in the processing of negative emotions elicited within certain social contexts. As such, our findings have potential clinical implications, paving the way for future clinicians to be able to accurately target specific brain regions among patients struggling with impaired social cognition abilities, including those diagnosed with posttraumatic stress disorder, autism spectrum disorder, social anxiety disorder, and depression.

## Abstract (precis)

The dorsolateral prefrontal cortex (DLPFC) and ventrolateral PFC (VLPFC) are both crucial structures involved in voluntary emotional regulation. However, it remains unclear whether the functions of these two cortical regions that are involved in emotional regulation, which are usually active in non-social situations, could be generalized to the regulation of social pain as well.

## Introduction

While the key brain regions for emotional regulation are primarily located in the prefrontal cortex (PFC; Ochsner et al., 2012), some studies have demonstrated that explicit emotional regulation using varying strategies do not involve the exact overlapping neural substrates (Morawetz et al., 2017; Vrtička et al., 2011). In particular, although the dorsolateral PFC (DLPFC) and ventrolateral PFC (VLPFC) are both important in distraction and reappraisal (Buhle et al., 2014; Kohn et al., 2014), e.g., the two regions often activate together during reappraisal (Ochsner et al., 2012; Morawetz et al., 2017), neuroimaging studies have revealed that while the VLPFC is consistently involved during reappraisal, the DLPFC is more associated with distraction (Dörfel et al., 2014; Moodie et al., 2020). However, it remains unclear to what extent these two brain regions are essential and specific for both of these strategies. This study employed transcranial magnetic stimulation (TMS) to examine the roles of the VLPFC and the DLPFC in emotional regulation via distraction and reappraisal strategies.

Meanwhile, a more urgent question is whether the brain regions involved in emotional regulation observed in general, especially non-social, situations can be generalized to functioning similarly in social contexts. Most previous studies have not disentangled the specific brain regions involved in emotional regulation for social versus non-social emotion-eliciting events (but see Vrtička et al., 2011). Uncovering the neural substrates critical for emotional regulation in social contexts is necessary not only for understanding the neural mechanisms behind emotional regulation, but also in the development of effective therapeutic protocols in clinics. For one thing, negative social experiences, including dysfunctional family interactions and traumatic or stressful interpersonal events, are all considered major risk factors in the development of psychiatric disorders such as posttraumatic stress disorder, social anxiety, depression, and autism spectrum disorder (Nolan et al., 2003; Durodié and Wainwright, 2019). For another, growing evidence indicates that these aforementioned psychiatric disorders are all associated with the maladaptive regulation of social pain (Davey et al., 2011; Masten et al., 2011). Therefore, it is implied that improving one's emotional regulation abilities in response to negative social events is highly beneficial for the remission of symptoms and recovery from psychiatric disorders (Laceulle et al., 2017). This study was designed to examine the causal relationship between the VLPFC/DLPFC and the emotional regulation of social pain to provide neural targets for clinical interventions.

Previous studies have demonstrated that both the VLPFC and the DLPFC are associated closely with the reduction of social pain (Koban et al., 2017a; Vijayakumar et al., 2017; Wang et al., 2017). Typically, researchers have observed that the right or bilateral VLPFC is activated significantly when individuals experienced social exclusion (Eisenberger et al., 2003; Masten et al., 2009; Onoda et al., 2010; Hooker et al., 2010; Riva et al., 2012, 2015; Hsu et al., 2015). Also, fMRI studies have found that enhanced activation in the DLPFC was associated with a decline in subjective social distress (Nishiyama et al., 2015; Koban et al., 2017b) and aggressive behaviors (Achterberg et al., 2016, 2020) following the experience of social rejection. However, while these studies have demonstrated an association between the DLPFC/VLPFC and reduced social pain, very limited studies have examined the role of these regions on explicit emotional regulation within social contexts (but see Koenigsberg et al., 2010). Recently, we used tDCS/TMS in an explicit emotional regulation task and provided direct evidence for the role of the right VLPFC on emotional regulation following the experience of social exclusion (He et al., 2018, 2020a,b). However, until now, no study has explored the causal role of the DLPFC in the explicit regulation of social pain. This study was thus inspired by this literature gap.

Our hypothesis is two-fold. First, the critical role of the VLPFC and the DLPFC in emotional regulation during non-social situations is generalizable to social ones, because previous studies have revealed a close association between the VLPFC/DLPFC and reduced social pain. Second, because the VLPFC is consistently involved during reappraisal and the DLPFC is always associated with distraction, we hypothesized that these two regions would show functional segregation, to some extent, for reappraisal and distraction strategies, respectively.

## Materials and Methods

### 

#### Participants

This study used three TMS groups: the VLPFC-activated group, the DLPFC-activated group, and the vertex-activated (sham) group. During the experiment design, we conducted a priori power analysis using G*Power 3.1.7 (*F* tests, ANOVA: repeated measures, within-between interaction) based on the effect size ($\eta_{p}^{2}$ = 0.130) reported in our previous TMS study (He et al., 2020a). According to the result of this power analysis, 18 participants in total would ensure 80% statistical power. However, six participants per group is such a small sample size in present-day neuroscience studies focusing on non-patient population. Thus, we finally decided to include 30 participants per TMS group, the same sample size as in our previous TMS study (He et al., 2020a), which ensured a statistical power near 100%. In line with our previous TMS study (He et al., 2020a), we decided to include 30 participants in each group. Therefore, a total of 90 healthy college students (all right-handed) were recruited from Shenzhen University. They completed five questionnaires before their group assignment, including the Trait form of Spielberger's State-Trait Anxiety Inventory (STAI-T; Spielberger et al., 1983), the Liebowitz Social Anxiety Scale (LSAS; Liebowitz, 1987), the Beck Depression Inventory Second Edition (BDI-II; Beck et al., 1996), the Rejection Sensitivity Questionnaire (RSQ; Downey and Feldman, 1996), and the Interpersonal Reactivity Index (IRI; Davis, 1980). The group assignments counterbalanced the scores of these questionnaires, with participants being assigned with equal numbers of males and females into the three TMS groups. No participant had any prior experiences with TMS before this experiment. No significant differences were found in participants' ages, or in their anxiety scores (STAI-T), social anxious levels (LSAS), depressive tendencies (BDI-II), rejection sensitivities (RSQ), or empathy (IRI) across all three groups (Table 1). The study protocol was approved by the Ethics Committee of Shenzhen University. Informed consent was signed by the participants before their engagement in the experiment.

**Table 1.** Demographical characteristics of the three groups (mean ± SD)

Items	VLPFC group (*n* = 30)	DLPFC group (*n* = 30)	Sham group (*n* = 30)	Statistics^a^	
				*F*	*p*
Gender (male/female)	15/15	15/15	15/15		
Age (year)	19.8 ± 1.6	19.2 ± 1.4	20.0 ± 1.8	1.71	0.186
STAI-T	40.8 ± 8.3	42.6 ± 10.9	39.1 ± 9.3	1.02	0.363
LSAS	36.5 ± 18.3	36.4 ± 17.6	38.4 ± 19.6	0.11	0.893
BDI-II	5.6 ± 4.1	6.9 ± 6.4	7.3 ± 7.1	0.66	0.522
RSQ	7.8 ± 3.7	7.8 ± 2.8	7.3 ± 3.6	0.26	0.775
IRI	52.7 ± 12.3	54.4 ± 10.3	53.7 ± 8.6	0.18	0.832

^a^ One-way ANOVA across the three groups.

STAI-T, the Trait form of Spielberger's State-Trait Anxiety Inventory; LSAS, the Liebowitz Social Anxiety Scale; BDI-II, the Beck Depression Inventory Second Edition; RSQ, the Rejection Sensitivity Questionnaire; IRI, the Interpersonal Reactivity Index.

#### Materials and experimental procedure

The experimental materials were 90 social exclusion pictures that were also used in our previous studies (He et al., 2018, 2020a,b). During the experiment, the images were presented in the center of an LCD screen with a viewing angle of 3.0 × 3.5°.

The study was a three (regulation type: no-regulation, reappraisal, and distraction) by three (TMS group: VLPFC-activated, DLPFC-activated, and sham) design. The regulation type was the within-subject factor and the TMS group was the between-subject factor. The task was divided into three blocks, corresponding to the three regulation types. To avoid any carry-over effects caused by the reappraisal/distraction instructions, the passive viewing (i.e., no-regulation) task was always performed first (see also He et al., 2018, 2020a,b). The 90 images were randomly assigned across the three blocks, with each block containing 30 images in total. The order of the three blocks was equal across the three TMS groups, while the order of the two emotional regulation blocks (reappraisal and distraction) was counterbalanced within each TMS group.

**Figure 1.**
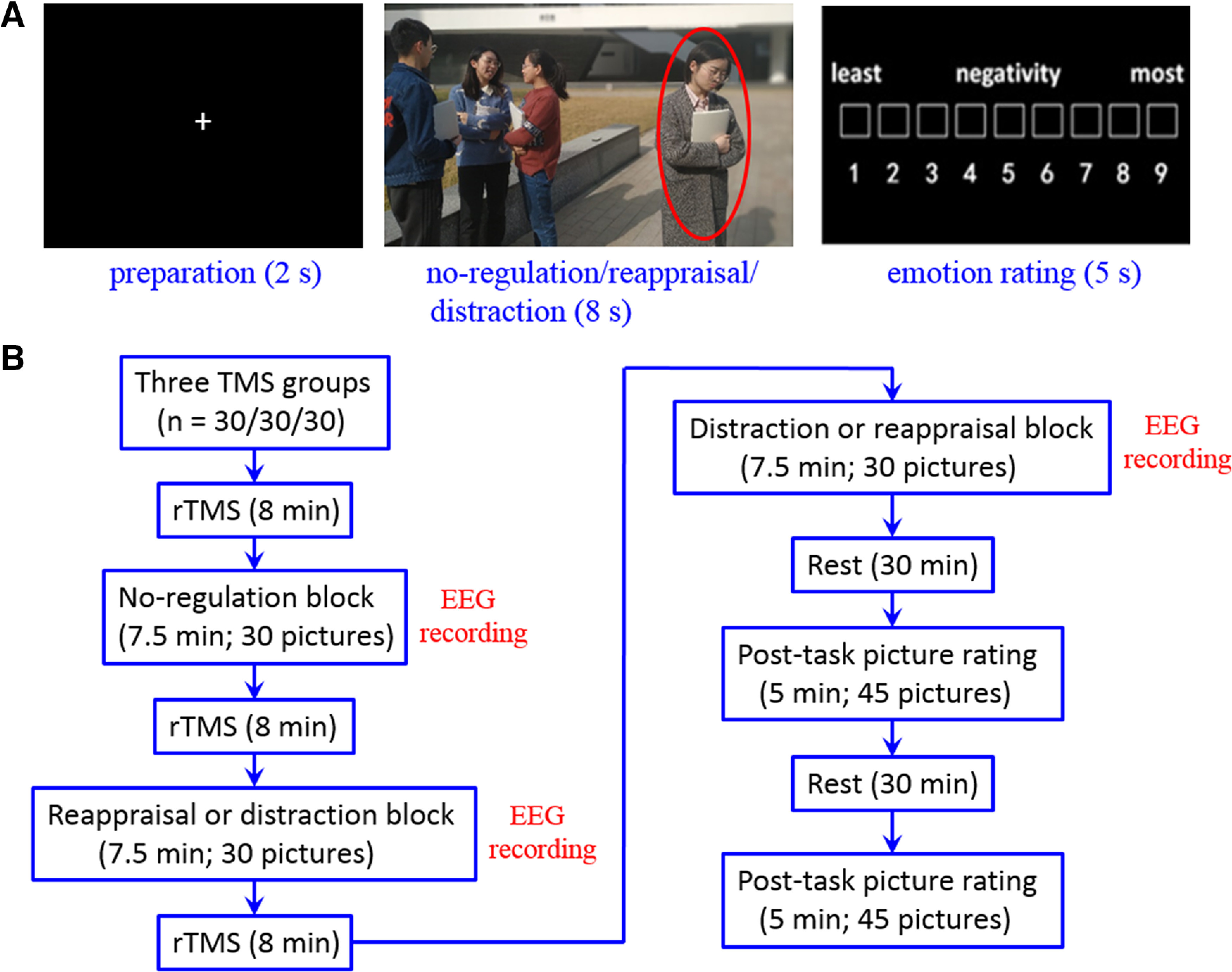
Experimental paradigm and sample images. ***A***, Stimulus presentation in one experimental trial. Because of copyright, the people in the sample image are replaced by graduate students from the research group. All four people in this picture gave their consent for this material to appear in academic journals. ***B***, Experimental procedure. Transcranial magnetic stimulation (TMS), repetitive TMS (rTMS), electroencephalogram (EEG).

As shown in Figure 1*A*, the trial began with a fixation (lasting 2 s), followed by the image presentation for 8 s, during which participants were required to either watch passively (during the no-regulation block) or to downregulate their negative emotions using reappraisal (during the reappraisal block) or to use distraction strategies (during the distraction block). They were then asked to report on the level of negative feelings they experienced on a nine-point scale (with a higher score indicating a higher level of negativity), by clicking the left button on the mouse.

At the start of the no-regulation block, participants were instructed as follows: “In this section, please think about how you would feel in a situation similar to that of the highlighted person in this picture.” At the start of the reappraisal block, participants were instructed as follows: “In this section, please imagine a better outcome or find a different explanation of the situation. For example, you could imagine that the group of people who are interacting with one another are talking about something that the person alone is not interested in or the person alone could make some change and join the group very soon. After you re-interpret the nature of this scene, please evaluate how you would feel in this situation if you were the highlighted person in the picture.” At the start of the distraction block, participants were instructed as follows: “In this section, please visually attend to the picture while producing unrelated neutral thoughts. For example, think about complex geometric designs or your next study plan. After you distract yourself from the picture, please assign a rating to your feelings in the situation as you were the highlighted person in the picture.”

The experimental procedure is shown in Figure 1*B*. Participants underwent three 8 min long TMS sessions during the experiment, with each TMS session occurring before each block. After the viewing and emotional regulation tasks, participants were allowed to relax for 30 and 60 min before rating the valence of the 90 pictures on a nine-point scale (1 representing the most negative valence, 5 for a neutral valence, and 9 for the most positive valence). For each participant, the 30 pictures per condition were randomly assigned to the 30- or 60-min valence rating task; that is, participants rated 45 pictures (15 per condition) during the 30-min rating task and the other 45 pictures during the 60-min rating task. The 45 pictures were randomly presented during these two rating tasks. The 30-min rating session was designed to repeat the result of our previous study (He et al., 2020a), while the 60-min rating session was designed to explore whether the prolonged-effect of TMS manipulation could be still detected 60 min after the task.

#### Repetitive TMS (rTMS)

**Figure 2.**
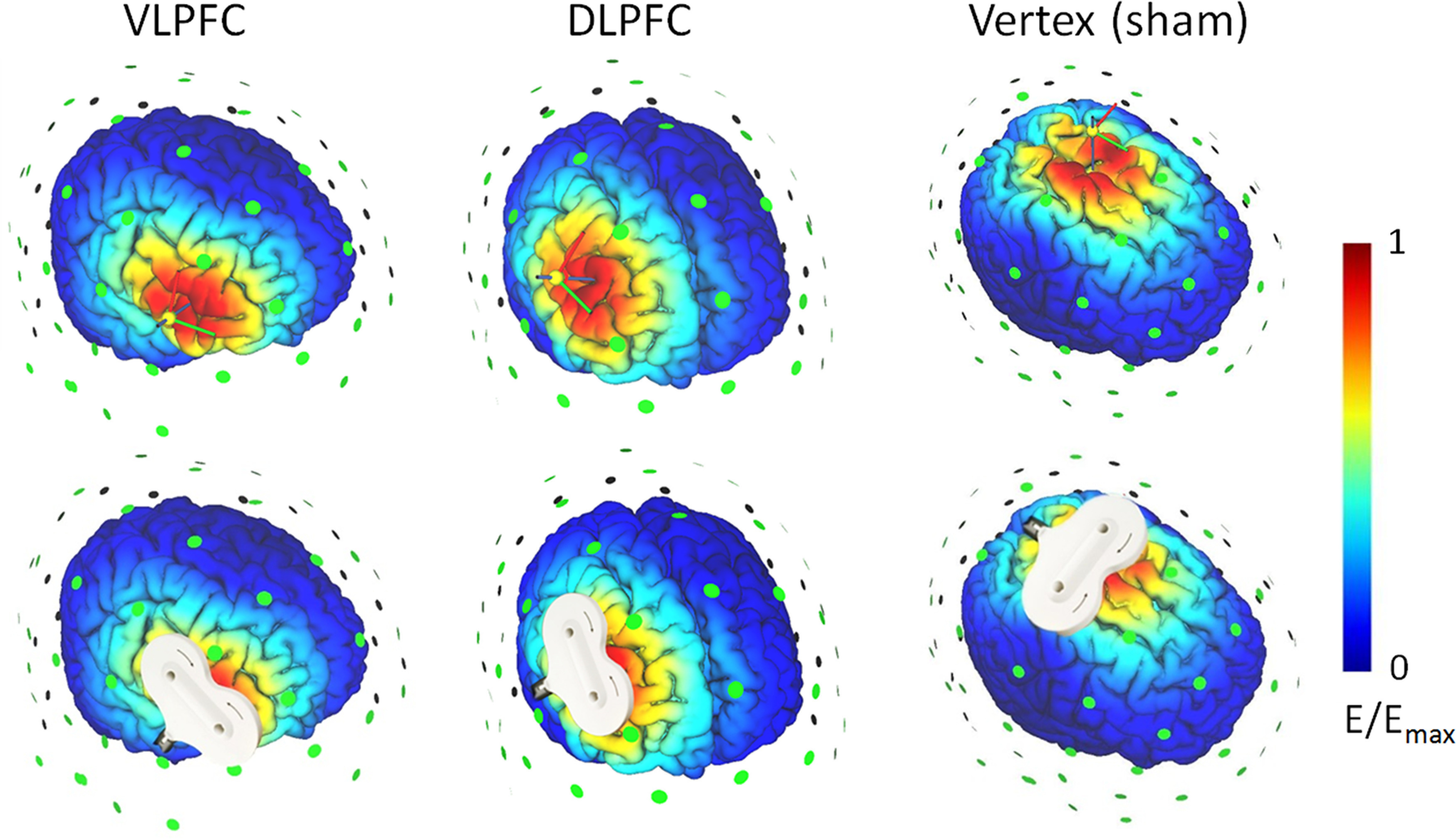
Illustration of TMS electric fields from the three TMS groups. The color represents the electric field strength, scaled from 0 (blue) to the individual maximums (red). The dorsolateral prefrontal cortex (DLPFC) and the ventrolateral prefrontal cortex (VLPFC).

This study used offline, instead of online, TMS to reduce any side effects that may have impacted participants' task performances (e.g., acoustic noise or muscle twitching). The TMS targets were the rVLPFC and rDLPFC for the two experimental groups. For the sham group, the TMS was targeted at the vertex so as to provide a similar scalp sensation as it did in the other two groups (Hartwright et al., 2016; Masina et al., 2019). A figure-eight-shaped coil was connected to the magnetic stimulator (M-100 Ultimate; Yingchi). The location of the coil was determined with reference to the International 10/20 electroencephalogram system (Klem et al., 1999). The rVLPFC is at the F8, the rDLPFC is at the F4, and the vertex is at the Cz. Each participant's resting motor threshold (rMT) was measured from their motor cortex (the C3), with the intensity being defined as 50% of the pulses that reliably produced thumb twitches (Schutter and van Honk, 2006). The rTMS was applied at 10 Hz at 90% of each participant's rMT. Each 8-min session contained 16 trains, with each train lasting for 3.9 s (a total of 624 pulses) and which were separated by intertrain intervals of 26.1 s (Raedt et al., 2010). The TMS-simulated electric field is illustrated on an adult brain model in Figure 2 (SimNIBS; www.simnibs.org).

#### EEG recordings and analysis

EEG data were recorded using a 32-channel amplifier (NeuSen.W32, Neuracle), with a sampling frequency of 250 Hz. Electrode impedances were kept below 10 kΩ. The reference electrode was placed at the CPz. No online filter was applied.

Data analysis was performed using MATLAB R2018a (MathWorks). Data were first re-referenced to the average of the left and right mastoids. Ocular artifacts were eliminated using the independent component analysis. Then, the EEG data were filtered using a 0.1- to 10-Hz bandpass filter with a slope of 24 dB/oct. The filtered data were segmented beginning 1 s before the onset of the picture and lasting for 9 s. The baseline-correction was based on the 1-s prestimulus time window. The Event-related potential (ERP) analysis focused on the late positive potential (LPP; Hajcak and Nieuwenhuis, 2006), which was measured as the average amplitude across the electrode sites at and around Pz (P3, P4, Pz, CP1, CP2, POz, PO3, and PO4). The time window for the LPP amplitude was chosen according to previous literature (Thiruchselvam et al., 2011; Schönfelder et al., 2014; Paul et al., 2016; Qi et al., 2017), beginning at the end of the typical P3 time window (500 ms) and lasting for the entire emotional regulation period (500–8000 ms after picture onset). Many previous studies have revealed that the downregulating of negative emotions, including social pain (Wang et al., 2017; He et al., 2020a), reliably reduces the LPP amplitudes (Hajcak et al., 2010; Liu et al., 2012).

#### Statistics

Statistical analysis was performed using SPSS Statistics 20.0 (IBM). Descriptive data are presented as mean ± SD, unless otherwise mentioned. Repeated-measures ANOVAs were performed on the subjective ratings of negative feelings and the LPP amplitudes, with regulation type (no-regulation, reappraisal, or distraction) as the within-subject factor and TMS group (VLPFC, DLPFC, or sham) as the between-subject factor. When analyzing the results of the pictures' valence ratings, a repeated-measures ANOVA was performed with testing time (30 or 60 min after the emotional regulation task) as the within-subject factor and TMS group as the between-subject factor. The Greenhouse–Geisser correction for the ANOVA tests was used whenever appropriate. A two-tailed Pearson's correlation was performed between the subjective ratings of negative feelings and the LPP amplitudes from the various regulation blocks and from the different groups. Multiple comparisons were corrected using the Bonferroni method.

## Results

### Ratings of negative emotions

The main effect of the regulation type was found to be highly significant (*F*_(2,174)_ = 303, *p* < 0.001, $\eta_{p}^{2}$= 0.777): participants reported fewer negative feelings in the reappraisal (3.0 ± 1.1) and distraction blocks (3.0 ± 1.0) when compared with the passive view (no-regulation) block (5.6 ± 1.1, *p*s < 0.001), whereas the negative feelings between the reappraisal and distraction blocks did not differ (*p* = 1.000). Additionally, there was a significant main effect of the TMS group (*F*_(2,87)_ = 7.08, *p* = 0.001, $\eta_{p}^{2}$ = 0.140): participants reported fewer negative feelings in the VLPFC (3.7 ± 1.6, *p* = 0.007) and DLPFC groups (3.6 ± 1.6, *p* = 0.003) when compared with the sham TMS group (4.3 ± 1.6), whereas the negative feelings between the two active TMS groups did not differ (*p* = 1.000).

**Figure 3.**
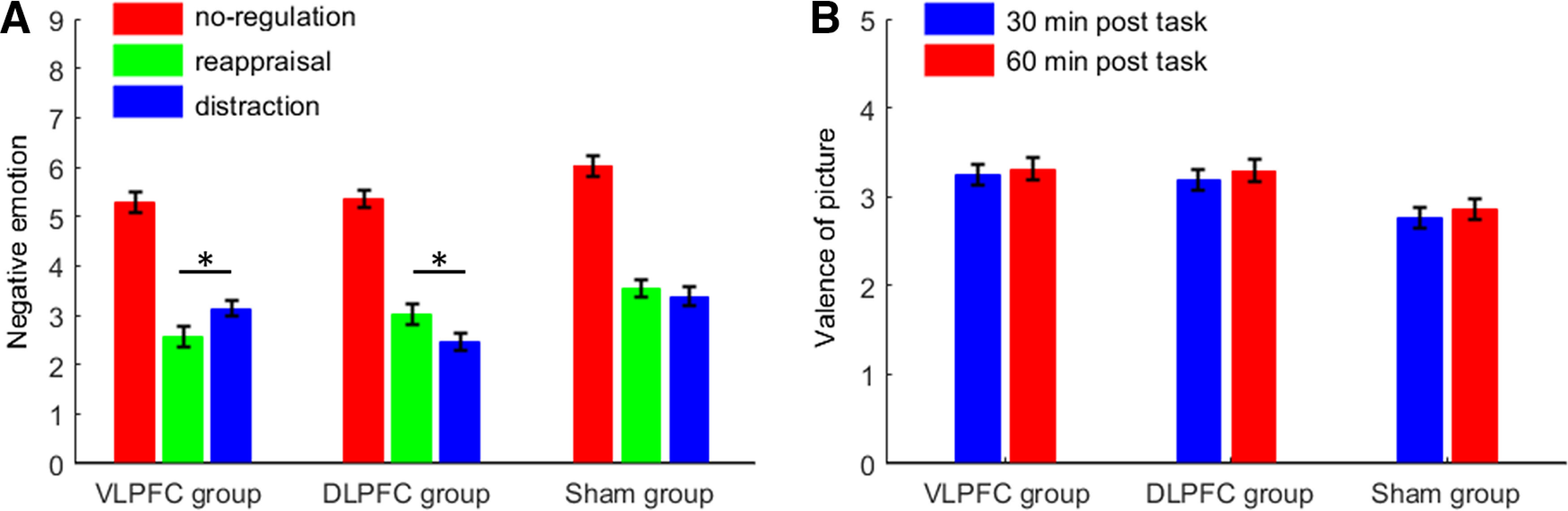
Ratings of negative emotions and picture valences. ***A***, Subjective ratings of negative emotions during the emotional regulation task. A nine-point scale was used, with higher scores indicating higher levels of negative emotions. ***B***, Postregulation ratings of the pictures' valences. A nine-point scale was used, with higher scores indicating more positive feelings toward the pictures (1 for most negative and 9 for most positive). Bars represent SEM; *p < 0.05.

More importantly, we observed a two-way interaction between TMS group × regulation type (*F*_(4,174)_ = 3.93, *p* = 0.005, $\eta_{p}^{2}$ = 0.083; Fig. 3*A*; Table 2). A simple effects analysis indicated that, while participants showed significantly reduced negative feelings in the reappraisal and distraction blocks when compared with the no-regulation one, across the three TMS groups (*p*s < 0.001), the two emotional regulation blocks showed different patterns of negative feelings across all groups. First, participants reported a slightly greater reduction of negative feelings in the reappraisal than in the distraction block in the VLPFC-activated group (*F*_(2,86)_ = 83.9, *p* < 0.001, $\eta_{p}^{2}$= 0.661; reappraisal vs distraction = 2.6 ± 1.2 vs 3.1 ± 0.8, *p* = 0.013). Second, contrarily, participants reported a slightly greater reduction in negative feelings during the distraction block than during the reappraisal one in the DLPFC-activated group (*F*_(2,86)_ = 97.4, *p* < 0.001, $\eta_{p}^{2}$ = 0.694; reappraisal vs distraction = 3.0 ± 1.1 vs 2.5 ± 0.9, *p* = 0.014). Third, no significant difference was observed between the reappraisal and distraction blocks for the sham group (*F*_(2,86)_ = 90.7, *p* < 0.001, $\eta_{p}^{2}$ = 0.678; reappraisal vs distraction = 3.6 ± 1.0 vs 3.3 ± 1.1, *p* = 1.000).

**Table 2.** Descriptive statistics of negative ratings and amplitudes of LPP component (mean ± SD)

Measure	TMS group	No-regulation	Reappraisal	Distraction
Negative emotion	VLPFC	5.28 ± 1.13	2.57 ± 1.16	3.13 ± 0.81
	DLPFC	5.35 ± 0.93	3.02 ± 1.07	2.46 ± 0.96
Sham	6.03 ± 1.14	3.54 ± 1.02	3.38 ± 1.09	
LPP amplitude (μV)	VLPFC	3.29 ± 2.92	0.72 ± 3.31	2.15 ± 2.92
	DLPFC	3.24 ± 2.68	2.05 ± 3.08	0.67 ± 3.28
	Sham	3.52 ± 2.40	1.92 ± 2.84	2.34 ± 3.14

### LPP amplitudes

The main effect of regulation type was found to be highly significant (*F*_(2,174)_ = 42.4, *p* < 0.001, $\eta_{p}^{2}$ = 0.328): participants showed smaller LPP amplitudes in the reappraisal (1.6 ± 3.1 μV) and distraction blocks (1.7 ± 3.2 μV) when compared with the no-regulation one (3.4 ± 2.6 μV, *p*s < 0.001), whereas the LPP amplitudes between the reappraisal and distraction blocks did not differ (*p* = 1.000). The main effect of the TMS group was not found to be significant (*F* < 1).

**Figure 4.**
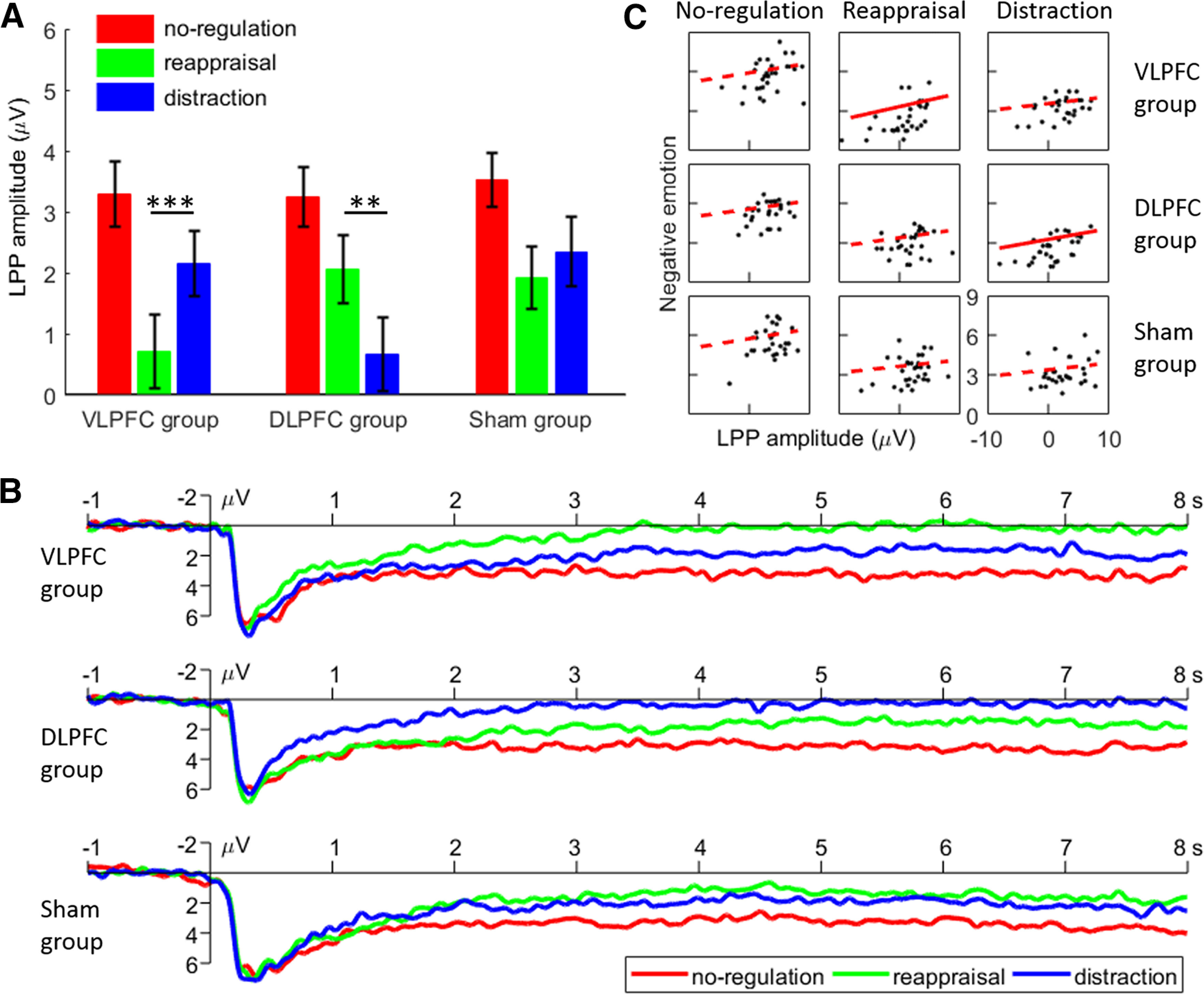
late positive potential (LPP) results. ***A***, The LPP amplitudes in the various conditions. Bars represent SEM; **p < 0.01, ***p < 0.001. ***B***, The grand-mean ERP waveforms for the three TMS groups. The data were averaged across P3, P4, Pz, CP1, CP2, POz, PO3, and PO4. ***C***, Correlations between LPP amplitudes and the subjective ratings of negative emotions. While the solid red line indicates a significant correlation, the dashed red line indicates a non-significant correlation.

More importantly, we observed a two-way interaction between TMS group × regulation type (*F*_(4,174)_ = 7.73, *p* < 0.001, $\eta_{p}^{2}$ = 0.151; Fig. 4*A*,*B*; Table 2). The simple effects analysis indicated that, while participants showed reduced LPP amplitudes in the reappraisal and distraction blocks when compared with the no-regulation one across the three TMS groups (*p*s ≤ 0.020), the two emotional regulation blocks showed differing patterns of LPP amplitudes across all three groups. First, participants showed smaller LPP amplitudes in the reappraisal than in the distraction block in the VLPFC-activated group (*F*_(2,86)_ = 29.9, *p* < 0.001, $\eta_{p}^{2}$ = 0.410; reappraisal vs distraction = 0.7 ± 3.3 vs 2.2 ± 2.9 μV, *p* < 0.001). Second, contrarily, participants showed smaller LPP amplitudes in the distraction than the reappraisal block in the DLPFC-activated group (*F*_(2,86)_ = 19.4, *p* < 0.001, $\eta_{p}^{2}$ = 0.311; reappraisal vs distraction = 2.0 ± 3.1 vs 0.7 ± 3.3 μV, *p* = 0.001). Third, no significant differences were observed between the reappraisal and distraction blocks in the sham group (*F*_(2,86)_ = 11.2, *p* < 0.001, $\eta_{p}^{2}$ = 0.207; reappraisal vs distraction = 1.9 ± 2.8 vs 2.3 ± 3.1 μV, *p* = 0.743).

### Correlations between LPP amplitudes and the subjective ratings of negative emotions

In each TMS group, we analyzed the correlations between the LPP amplitudes and the ratings of negative emotions in the three regulation blocks, separately. For example, the LPP amplitudes in the reappraisal block were correlated with the rating of negativity in this block for each group (*n* = 30). This procedure produced nine correlations across the three TMS groups (Fig. 4*C*; Table 3). After correcting for multiple tests, the LPP amplitudes and the ratings of negativity were found to be significantly correlated within the reappraisal block for the VLPFC group (*r* = 0.570, corrected *p* = 0.009), as well as during the distraction block for the DLPFC group (*r* = 0.580, corrected *p* = 0.007).

**Table 3.** Correlation statistics between LPP amplitudes and subjective ratings of negativity (*n* = 30)

Regulation type	VLPFC group			DLPFC group			Sham group		
	*r*	*p*	*p*_cor_^a^	*r*	*p*	*p*_cor_^a^	*r*	*p*	*p*_cor_^a^
No-regulation	0.347	0.060	>0.05	0.297	0.110	>0.05	0.346	0.061	>0.05
Reappraisal	0.570	< 0.001	0.009**	0.373	0.042	>0.05	0.251	0.182	>0.05
Distraction	0.338	0.068	>0.05	0.580	<0.001	0.007**	0.310	0.095	>0.05

^a^ Corrected using Bonferroni method;

**p < 0.01.

### Posttask valence ratings of the pictures

The main effect of the TMS group was found to be significant (*F*_(2,87)_ = 5.6, *p* = 0.005, $\eta_{p}^{2}$= 0.114; Fig. 3*B*), the valences reported by both the VLPFC-activated (3.3 ± 0.6, *p* = 0.011) and the DLPFC-activated groups (3.2 ± 0.6, *p* = 0.020) were higher than those reported by the sham TMS group (2.8 ± 0.6). Neither the main effect of testing time (*F*_(1,87)_ = 3.5, *p* = 0.066, $\eta_{p}^{2}$= 0.038; 30 min vs 60 min = 3.1 ± 0.7 vs 3.2 ± 0.7) nor the interaction between the TMS group and testing time were significant (*F* < 1).

## Discussion

This study employed TMS to causally explore the neural substrates of explicit emotional regulation, using reappraisal and distraction strategies, within social contexts. We used the subjective rating of negative emotions and the objective ERP index of LPP amplitudes to measure emotion regulation effects. Our results demonstrate that (1) both of the lateral PFC (LPFC) regions within the right hemisphere highly facilitate social pain relief (reflected by lower negative emotional ratings and reduced LPP amplitudes), and (2) while the TMS-activated VLPFC had a better regulation effect during the reappraisal condition, the TMS-activated DLPFC had a better regulation effect during the distraction one.

We focused on the DLPFC and the VLPFC, as they have both been established as key cortical regions involved in emotional regulation (Ochsner et al., 2012; Dörfel et al., 2014; Kohn et al., 2014; Morawetz et al., 2017). For one thing, the neural model of emotional processing (Etkin et al., 2015) proposes that, while automatic or implicit emotional regulation predominantly involves the medial PFC (MPFC) system, voluntary or explicit emotional regulation primarily involves the LPFC system. For another, it has been demonstrated that, when compared with the MPFC, the LPFC is more frequently found impaired among psychiatric disorders (Minzenberg et al., 2009; Hamilton et al., 2012) and in individuals experiencing suicidal thoughts (Schmaal et al., 2020). For example, depressed patients tend to show hypoactive LPFCs during voluntary emotional control, whereas their MPFC often functions well during automatic emotional regulation (Rive et al., 2013). These findings suggest that the LPFC, instead of the MPFC, is the ideal target for TMS or neurofeedback therapies aimed at improving psychiatric patients' voluntary emotional regulation abilities. This study contributes to the psychiatric field by causally demonstrating that the two LPFC regions are able to successfully downregulate negative emotions caused by not only non-social but also social events.

Our findings have some potential implications for clinical practice; that is, the right VLPFC/DLPFC regions are valid, and possibly the most direct brain targets, for the treatment of socially-based emotional dysregulation. Previous studies have shown that, across various clinical populations, patients have often demonstrated reduced recruitment of the VLPFC and the DLPFC during the downregulation of negative emotions (Phillips et al., 2008; Erk et al., 2010; Rive et al., 2013; Zilverstand et al., 2017; Park et al., 2019). More relevant to our work, studies have found that depressed patients demonstrate reduced activation in their right VLPFCs in response to social exclusion images when compared against healthy controls (Elliott et al., 2012), and that enhanced LPFC engagement during negative emotional regulation predicted a decrease in depression severity over six months (Heller et al., 2013). Beyond the findings of these previous studies, our work further reveals the causal role of the VLPFC/DLPFC on social pain relief, which provides a clear rationale for targeting these two areas in the treatment of patients with deficits in social emotional regulation.

The most novel finding of this study is that distraction and reappraisal strategies show double dissociation to a certain extent. This result is in line with the cognitive model of emotional regulation (Gross, 1998), which states that these two regulation strategies function separately during the early and late stages of peoples' cognitive control of their emotions. Additionally, this finding is consistent with previous fMRI observations, demonstrating that distraction and reappraisal, specifically and separately, recruit the DLPFC and the VLPFC, respectively (Dörfel et al., 2014; Moodie et al., 2020). The contribution of this study is two-fold. First, we used a manipulation technique to separate the regulatory functions of the DLPFC and the VLPFC across two groups so as to provide causal evidence beyond that of previous neuroimaging findings. Second, this study found that the LPP amplitudes during the reappraisal and distraction conditions were specifically correlated with the negative ratings reported by the VLPFC-activated and the DLPFC-activated groups. These results provide novel electrophysiological evidence for the relative specificity of the VLPFC and the DLPFC during emotional regulation in the use of reappraisal and distraction strategies, respectively.

Previous studies have established that the DLFPC plays a fundamental role in peoples' cognitive control abilities (Crone and Steinbeis, 2017) and is a key region involved in the attentional network (Zwanzger et al., 2014). Given that the core cognitive process of distraction is essentially an attentional shift (Ochsner and Gross, 2005), the DLPFC is frequently implicated as a critical brain region that manages a person's attention during the early stages of emotional regulation (Kohn et al., 2014; Zwanzger et al., 2014). The DLPFC has been shown to indirectly inhibit subcortical limbic structures, such as the amygdala (Park et al., 2019). In particular, the DLPFC communicates with the dorsal ACC/MPFC to monitor conflict and signal the need for behavioral change. The DLPFC also communicates with the parietal region to signal the latter to allocate one's attention elsewhere. These two pathways associated with the DLPFC during emotional regulation result in the downregulation of emotional responses from the ventral ACC/MPFC-amygdala network (Ochsner and Gross, 2005; Mitchell, 2011; Iordan et al., 2019). It has been established that the DLPFC is highly impaired in various psychiatric disorders (Hamilton et al., 2012; Glausier and Lewis, 2018). For example, hypoactivity within this cortical region was observed during attentional control in either emotional regulation or distractor inhibition tasks among patients with depression (Fales et al., 2008) and anxiety (Bishop, 2009).

Unlike the DLPFC, many studies have found that the VLPFC is the primary region involved in reappraisal strategies (Price et al., 2013; Dörfel et al., 2014). It achieves regulatory goals by selecting goal-consistent responses using one's semantic memory and inhibits goal-inconsistent responses so as to reinterpret the affective stimuli (Wager et al., 2008; Ochsner et al., 2009, 2012). In particular, the VLPFC modulates emotional processing through direct projections to the ventral MPFC-amygdala pathway (Silvers et al., 2017), while it also inhibits undesirable responses through projections to both the ACC and the insula (Kohn et al., 2014). Relevant to the current work, a recent study using tDCS demonstrated that successful achievement of emotional reappraisal was based primarily on the VLPFC rather than on the DLPFC (Marques et al., 2018). Taken together, the findings of previous studies in conjunction with our own provide converging evidence that the VLPFC plays a critical role in emotional regulation using reappraisal strategies.

The findings that the VLPFC and the DLPFC play relatively specific roles in different regulation strategies are valuable for clinical practice, which might help to refine the targeting of brain areas in future treatment protocols. For example, anxiety and depressive symptoms are associated with a less frequent use of reappraisal strategies when compared with healthy controls (Dryman and Heimberg, 2018). Meanwhile, the specific strategy that one employs also depends on certain contextual information, for example, distraction is more frequently used for high-intensity stimuli, whereas reappraisal is preferred following low-intensity stimuli (Sheppes et al., 2011; Shafir et al., 2015). We thus propose that TMS or neurofeedback therapies should target the most accurate brain region based on each patient's cognitive characteristics, symptoms, or trigger factors so as to effectively enhance their unique emotional regulation abilities.

Another encouraging finding is that the TMS-induced effect of social pain relief persisted for more than 1 h, as revealed by more positive valances reported at both 30 and 60 min following emotional regulation by the two active TMS groups when compared with the sham group. This result not only corroborates those of previous studies that found that the TMS-activated VLPFC reduces social pain after 0.5 h following emotional reappraisal (He et al., 2020a), but also extends the prolonged-effect of the TMS in social emotional regulation to at least 1 h. These findings are consistent with those of prior studies demonstrating that the prolonged-effect of a single rTMS session (averaging 20–25 min) usually lasts for 30–60 min (Valero-Cabré et al., 2017). However, we should have a cautious attitude toward the translation of the current finding to a clinical context, because this study only examined healthy people and the prolonged-effect of 1 h is far from enough in clinics. Future studies should test the prolonged-effect in clinical patients and try to establish multiple-session protocols so as to maintain these TMS-induced neural plastic changes over a longer term.

Finally, it should be noted that we examined imagined, rather than actual, social pain. Although this imagining paradigm has been proven efficient manner in evoking negative emotions and assessing emotional regulation effects (Ochsner et al., 2002, 2004; Wager et al., 2008), it relies on an empathy-based process that might introduce certain confounding factors. Therefore, we strongly encourage future studies to verify these current findings using paradigms evoking “first-hand” social pain; for example, using Cyberball, Island Getaway, or other similar methods. Additionally, this study focused on the right hemisphere of the VLPFC/DLPFC based on the work of previous studies (Ochsner et al., 2012; Morawetz et al., 2017; He et al., 2018, 2020a,b). Future work should test and compare the left versus right hemispheric effects in both healthy individuals and those diagnosed with certain psychiatric disorders. Last but not least, we speculated in this study that a TMS session with 10 Hz stimuli could activate brain regions according to a majority of TMS literature (Rossi and Rossini, 2004; Dayan et al., 2013). In order to confirm the current conclusion, it is preferred to add another three groups using an inhibited TMS manipulation (e.g., with 1 Hz) in future work.

In summary, both the behavioral and electrophysiological results of this study support the hypotheses that both the VLPFC and the DLPFC play causal roles in peoples' explicit emotional regulation of social pain, and that these two regions show relative functional specificity for reappraisal and distraction strategies. In addition, the TMS effect was observed to be sustainable for at least 1 h. These findings pave the way for the accurate targeting of the VLPFC and/or the DLPFC to improve the social functioning and emotional regulation abilities of people within clinical populations.
